# Role of Peroxisome Proliferator-Activated Receptor-***γ*** in Vascular Inflammation

**DOI:** 10.1155/2012/508416

**Published:** 2012-07-24

**Authors:** Kousei Ohshima, Masaki Mogi, Masatsugu Horiuchi

**Affiliations:** Department of Molecular Cardiovascular Biology and Pharmacology, Graduate School of Medicine, Ehime University, Shitsukawa, Ehime, Toon 791-0295, Japan

## Abstract

Vascular inflammation plays a crucial role in atherosclerosis, and its regulation is important to prevent cerebrovascular and coronary artery disease. The inflammatory process in atherogenesis involves a variety of immune cells including monocytes/macrophages, lymphocytes, dendritic cells, and neutrophils, which all express peroxisome proliferator-activated receptor-**γ** (PPAR-**γ**). PPAR-**γ** is a nuclear receptor and transcription factor in the steroid superfamily and is known to be a key regulator of adipocyte differentiation. Increasing evidence from mainly experimental studies has demonstrated that PPAR-**γ** activation by endogenous and synthetic ligands is involved in lipid metabolism and anti-inflammatory activity. In addition, recent clinical studies have shown a beneficial effect of thiazolidinediones, synthetic PPAR-**γ** ligands, on cardiovascular disease beyond glycemic control. These results suggest that PPAR-**γ** activation is an important regulator in vascular inflammation and is expected to be a therapeutic target in the treatment of atherosclerotic complications. This paper reviews the recent findings of PPAR-**γ** involvement in vascular inflammation and the therapeutic potential of regulating the immune system in atherosclerosis.

## 1. Introduction

Atherosclerosis is the primary cause of cerebrovascular and coronary artery disease through slowly progressive lesion formation and luminal narrowing of arteries. This vascular remodeling leads to thrombotic complications including acute coronary syndrome, myocardial infarction, and stroke. Atherosclerosis is well known to be an inflammatory disease, and the underlying pathology is characterized by a persistent inflammatory process of the arterial wall [1]. With increasing prevalence of risk factors such as hypertension, diabetes, and obesity [2], it is critical to control vascular inflammation in order to decrease mortality and improve public health. To solve this problem, peroxisome proliferator-activated receptor (PPAR)-*γ* has emerged as an important player.

PPAR-*γ* belongs to the nuclear receptor family of ligand-activated transcription factors, which also include the steroid and thyroid hormone receptors [3]. PPAR-*γ* forms heterodimers with the retinoid X receptor (RXR) and activates transcription by binding to a specific DNA element known as the PPAR response element (PPRE) [4]. In the absence of ligand, PPAR-RXR heterodimers bind a number of corepressors, including nuclear receptor corepressor and the silencing mediator of the retinoid and thyroid hormone receptors, to suppress the target genes. In the presence of selective ligands, PPAR-*γ* undergoes a conformational change facilitating the dissociation of corepressors and the recruitment of co-activators, leading to transcriptional activation of the target genes [5, 6]. To date, a variety of endogenous and synthetic ligands in addition to its co-activators have been detected (Table 1). PPAR-*γ* is known to have four splice isoforms: PPAR-*γ*1, *γ*2, *γ*3, and *γ*4. PPAR-*γ*1 and *γ*2 have been identified in mouse, whereas in humans and other species, at least two other isoforms, PPAR-*γ*3 and *γ*4, have also been detected [7]. PPAR-*γ*3 and *γ*4 encode the same protein as PPAR-*γ*1, which is expressed in endothelial cells (ECs), vascular smooth muscle cells (VSMCs), macrophages, and cardiomyocytes. On the other hand, PPAR-*γ*2 is mainly expressed in adipocytes [8].

**Figure 1:**
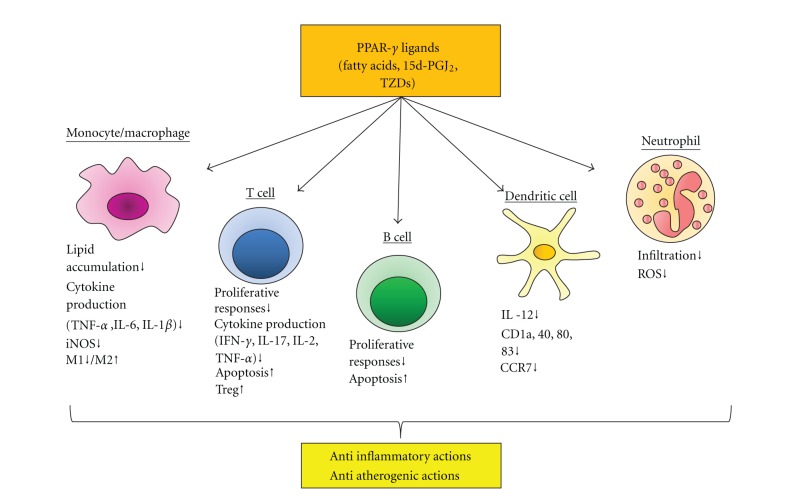
Effects of PPAR-*γ* activation on various immune cells in vascular inflammation. PPAR-*γ* is expressed in various immune cells such as monocyte/macrophage, lymphocyte, dendritic cell, and neutrophil. PPAR-*γ* activation by endogenous and synthetic ligands could regulate inflammatory responses induced by these cells, leading to anti-inflammation and antiatherogenicity. CCR, chemokine (C-C motif) receptor; 15d-PGJ2, 15-deoxy-Δ^12, 14^-prostaglandin J_2_; IFN, interferon; IL, interleukin; ROS, reactive oxygen species; TNF, tumor necrosis factor; Treg, regulatory T cell; TZD, thiazolidinedione.

PPAR-*γ* plays an important role in regulation of adipocyte differentiation and insulin resistance [9]. The thiazolidinedione (TZD) class of synthetic PPAR-*γ* ligands reduces peripheral insulin resistance and has been widely used to treat type 2 diabetes mellitus. For instance, several reports using high-fat diet-induced obese mice demonstrated that PPAR-*γ* agonists had beneficial effects on improving insulin resistance and inflammation [10–13]. In addition, recent large clinical studies have demonstrated that a PPAR-*γ* agonist had beneficial effects not only on glycemic control but also in preventing atherosclerotic disease [14–17]. The lines of evidence derived from study of EC specific PPAR-*γ* null mice [18–20] and from virus-mediated constitutive expression of PPAR-*γ* in human ECs [21] have also shown important roles of PPAR-*γ* on atherogenesis. Increasing evidence has demonstrated that PPAR-*γ* plays important roles in the immune system, since PPAR-*γ* is expressed in inflammatory cells such as macrophages, T cells, B cells, and dendritic cells [22]. These results suggest that PPAR-*γ* activation is an important regulator in vascular inflammation and is expected to be a therapeutic target in the treatment of atherosclerotic complications (Figure 1). The present paper focuses on the role of PPAR-*γ* in vascular inflammation beyond its beneficial effects on glycemic control and discusses the potential therapeutic roles of regulating PPAR-*γ* activation.

## 2. PPAR-**γ** and Monocytes/Macrophages

Monocytes/macrophages are key players in vascular inflammation and atherosclerosis [23]. PPAR-*γ* has been detected in rodent macrophages [24], and human macrophages in atherosclerotic lesions [25]. Differentiated macrophages show two acquired phenotypic characteristics, the classically activated (M1) phenotype and the alternatively activated (M2) phenotype [26]. M1 activation is triggered by stimulation such as by bacterial lipopolysaccharide (LPS) and is associated with the production of proinflammatory cytokines including interferon-*γ* (IFN-*γ*) and interleukin-12 (IL-12), which are linked to T helper 1 (Th1) immune responses. In contrast to M1, M2 activation is triggered by IL-4 and IL-13, which are linked to Th2 responses [27]. M1 macrophages produce a number of proinflammatory cytokines and express a high level of reactive oxygen species (ROS), having antimicrobial activity. On the other hand, M2 macrophages generate anti-inflammatory products and are involved in pathogen sequestration, wound healing, and phagocytosis of apoptotic cells [28, 29]. The balance between these two subsets is thought to be important in regulating vascular inflammation.

*In vitro* studies have demonstrated that PPAR-*γ* agonists attenuated the gene expression and secretion of proinflammatory cytokines associated with M1 macrophages in human monocytes, such as tumor necrosis factor-*α* (TNF-*α*), IL-1*β*, and IL-6 [30], and reduced the activity of macrophages including the transrepression of nuclear factor kappa B (NF-*κ*B) [24]. In addition, troglitazone and rosiglitazone, PPAR-*γ* agonists, inhibited monocyte chemotactic protein 1 (MCP-1)-directed monocyte migration through modulation of matrix metalloproteinase-9 (MMP-9) and tissue inhibitor of matrix metalloproteinase-1 (TIMP-1) production [31]. These results suggest that PPAR-*γ* activation may be involved in vascular inflammation through regulating macrophage activation.

PPAR-*γ* has been also reported to be an invaluable transcriptional regulator of monocyte phenotypic differentiation. Crosstalk between PPAR-*γ* and IL-4 signaling is thought to be important for M2 macrophage polarization [32, 33]. In macrophages, IL-4-mediated signaling activates the transcription factor, signal transducers, and activators of transcription 6 (STAT6), resulting in upregulation of the expression of PPAR-*γ*, PPAR-*γ* coactivator-1*β* (PCG-1*β*), and ARG1. Increased PCG-1*β* enhances STAT6 action on these genes and other genes relating to mitochondrial biogenesis, oxidative metabolism, and M2 differentiation. Additionally, other recent studies have demonstrated that PPAR-*γ*-deficient macrophages were resistant to M2 polarization and promoted insulin resistance [29, 34].

Foam cell formation of macrophages is also important in the progression of atherosclerosis. Another function of PPAR-*γ* in macrophages is regulation of lipoprotein uptake and cholesterol efflux. Tontonoz et al. have demonstrated that PPAR-*γ* ligands induced the differentiation of human monocytes into macrophages and enhanced the transcription of a scavenger receptor for oxidized low-density lipoprotein (oxLDL), CD36 [35]. In addition, oxidized lipids inside the oxLDL particle, including 9-hydroxyoctadecadienoic acid (9-HODE) and 13-HODE, enhance PPAR-*γ* activation [36]. Thus, PPAR-*γ* activation in the presence of oxidized lipids could lead to a positive feedback loop to promote foam cell formation [37, 38]. On the other hand, *in vivo* studies revealed that TZD treatment could increase macrophage CD36 expression, but did not enhance foam cell formation, suggesting that PPAR-*γ* could activate other pathways that enhance cholesterol efflux and reduce intracellular cholesterol level. The enhancement of cholesterol efflux was mediated by the cholesterol-phospholipid transporter ABCA1, which is an indirect target gene of PPAR-*γ* via liver X receptor *α* [39, 40]. These results suggest that PPAR-*γ* activation couples oxLDL uptake to cholesterol efflux, thus enhancing the removal of oxLDL from the vessel wall.

## 3. PPAR-**γ** and T Cells

PPAR-*γ* is expressed in T cells, and its expression is increased in activated T cells [41]. It is reported that PPAR-*γ* activation modulates the expression of proinflammatory Th1 cytokines in CD4-positive lymphocytes. For instance, 15-deoxy-Δ^12, 14^-prostaglandin J_2_ (15d-PGJ_2_), an endogenous PPAR-*γ* ligand, and TZDs reduced IL-2 secretion from murine T cell clones [42] and inhibited IL-2 production and phytohemagglutinin-inducible proliferation in human T cells in a dose-dependent manner [43]. In addition, PPAR-*γ* activators inhibited the expression of proinflammatory cytokines such as interferon-*γ* (IFN-*γ*), TNF-*α*, and IL-2, leading to attenuation of human monocyte CD64 expression and human endothelial cell major histocompatibility complex class II induction [44]. In a well-established mouse colitis model, it is reported that TZDs attenuated intestinal inflammation, at least in part, due to immune deviation away from Th1 and towards Th2 cytokine production [45].

Th17 cells and a proinflammatory cytokine, IL-17, secreted by them have been reported to be involved in the pathogenesis of atherosclerotic disease. Recently, Klotz et al. have indicated that PPAR-*γ* activation can regulate the differentiation and function of Th17 cells, a newly identified T cell subset [46]. PPAR-*γ* activators could suppress the differentiation of Th17 cells by inhibiting the upregulation of retinoic acid receptor-related orphan receptor *γ*t (RoR*γ*t), the key transcriptional factor of Th17 differentiation, in response to Th17 cell-promoting cytokines, such as TGF-*β* and IL-6. Therefore, PPAR-*γ* activation selectively suppressed Th17 cell differentiation, but not the differentiation of Th1, Th2, or regulatory T cells (Treg). Pharmacologic activation of PPAR-*γ* prevented removal of the silencing mediator for retinoid and thyroid hormone receptors corepressor from the RoR*γ*t promoter in T cells, thus interfering with RoR*γ*t transcription. Both T cell-specific PPAR-*γ* knockout and endogenous ligand activation revealed the physiological role of PPAR-*γ* in continuous T cell intrinsic control of Th17 differentiation.

CD4+CD25+ Tregs also play an important role in the pathogenesis of atherosclerosis and are expected to be a novel therapeutic target to attenuate atherosclerosis and stabilize vulnerable plaques [47]. A relationship between PPAR-*γ* activation and regulation of Tregs has been reported. The PPAR-*γ* ligand, ciglitazone, enhanced the conversion of effector T cells to Tregs *in vitro* and had an enhancing effect on both natural and inducible Tregs [48]. Moreover, Lei et al. have demonstrated that PPAR-*γ* activation with endogenous and synthetic ligands together with transforming growth factor-*β* (TGF-*β*) elicited Foxp3 deoxyribonucleic acid (DNA) methylation through potent downregulation of DNA methyltransferases (DNMTs) such as DNMT1, 3a, and 3b, and induced potent and stable Foxp3, resulting in the generation of functional inducible Tregs [49].

## 4. PPAR-**γ** and B Cells

B cells play an important role in atherosclerosis and are thought to have atherogenic and antiatherogenic effects according to their subtype [50]. Mature B cells are categorized into three subtypes according to their surface antigens: conventional B2 B cells, B1 B cells, and marginal zone B cells [51]. The conventional B2 B cell plays an important role in adaptive immunity by producing specific antibodies to cognate antigens. The B1 B cell, which is found primarily in serosal cavities such as the peritoneal and pleural cavities, is important in innate immunity and responsible for production of natural IgM antibodies. The marginal zone B cell in splenic tissue plays a role in first-line defense against circulating blood-borne antigens. B1 B cells are thought to have a protective effect against atherogenicity [52, 53]. On the other hand, it seems that B2 B cells are involved in atherosclerosis, since native conventional B2 B cells can differentiate into two effector B cells, so-called Be1 and Be2 B cells. Be1 B cells produce Th1 cytokines including INF-*γ*, IL-2 and IL-12, whereas Be2 B cells secrete IL-4, IL-6 and IL-10, which are Th2 cytokines. It is reported that these cytokines secreted by Be cells enhance immunomodulation during chronic inflammation [54]. However, the detailed role of Be1 and Be2 B cells in atherosclerosis remains to be elucidated. Recently, regulatory B cells that produce IL-10 have been recognized as an important component of the immune system [55–59]. Regulatory B cells secrete IL-10, and this may lead to suppression of both Th1 and Th2 polarization and inhibition of proinflammatory cytokine production from macrophages and DC. The role of regulatory B cells in atherosclerosis also remains to be elucidated, but they may attenuate the progression of atherosclerosis.

PPAR-*γ* is expressed in human and mouse B cells [60, 61]. Most studies of the effect of PPAR-*γ* activation on B cells focus on the apoptotic effect of endogenous and synthetic ligands on normal or B lymphoma cells. Recent reports demonstrated that detailed roles of PPAR-*γ* and RXR*α* agonists in PPAR-*γ* agonist-induced apoptosis of B cells were activation of mitogen-activated protein kinases (MAPKs), inhibition of nuclear factor-kappa B (NF-*κ*B), and CD40 activation [62–66]. On the other hand, a recent paper by Garcia-Bates et al. reported the role of the PPAR-*γ*/RXR*α* pathway in human B cell differentiation [67]. They demonstrated that activated B cells have upregulated expression of PPAR-*γ*. In addition, nanomolar levels of PPAR-*γ* ligands, such as 15d-PGJ_2_ and rosiglitazone, enhanced B cell proliferation and significantly stimulated plasma cell differentiation and antibody production. The simultaneous addition of nanomolar concentrations of the RXR*α* ligand, 9-*cis*-retinoic acid, and PPAR-*γ* ligands to CpG-activated B cells resulted in additive effects on B cell proliferation, plasma cell differentiation, and antibody production. This result suggests that PPAR-*γ* activation may also regulate the function and differentiation of B cells. However, the link between PPAR-*γ* activation and B cell function in atherosclerosis is still unclear.

## 5. PPAR-**γ** and Dendritic Cells

DC contributes to chronic vascular inflammation, leading to atherosclerosis and its complications [68–70]. In fact, a number of DC has been observed in atherosclerotic lesions of mouse models [71–73] and in human advanced plaques [74–76]. In normal conditions, DC is professional antigen-presenting cell that presents many kinds of endogenous and exogenous antigens to T cells, providing an important link between innate and adaptive immune responses [77]. Additionally, many lines of evidence have demonstrated that DC contributes to the pathogenesis and progression of atherosclerosis [68–70]. DC accumulates in the intima of atherosclerotic lesions through vascular cell adhesion molecule-1 (VCAM-1) and CX3C chemokine receptor 1 (CX3CR1) during low-grade chronic inflammation [72, 78]. DC may differentiate from Ly6^low^ monocytes that CX3CR1-dependently patrol arterial vessels, but can also differentiate from Ly6^high^ monocytes, which seem to be preferentially recruited. In intimal proliferation of DC, granulocyte macrophage colony-stimulating factor (GM-CSF) is thought to be important [79, 80]. Excess lipoproteins deposited in the arterial wall accumulate within lipid-loaded CD11c^+^ DC, contributing to early-stage plaque formation. DC can control lipid homeostasis possibly through lipoprotein uptake and clearance from the circulation. DC also regulates T cell activation in the vessel wall and influence helper T cell responses, with lipoprotein being able to contribute to DC maturation and activation [69]. In addition, various DC subsets can release proinflammatory cytokines [68]. For instance, conventional DC (cDC) can participate in interaction with T and natural killer T cells, which results in increased production of IFN-*γ*, IL-17, and TNF-*α* from T cells [81]. Activation of CD36 and Toll-like receptors (TLRs) in CD11b+CD11c+ DC and cDC by lipids results in increased secretion of various DC-derived cytokines, such as IL-6, IL-12, and TNF-*α* [82]. Plasmacytoid DC (pDC) has been shown to produce amounts of type I IFNs (IFN-*α* and *β*), which play a proatherogenic role.

PPAR-*γ* is expressed in murine and human DC, and PPAR-*γ* activation has been shown to be involved in DC function [83–90]. PPAR-*γ* ligands inhibited the production of IL-12 and several cytokines such as chemokine (C-X-C motif) ligand 1 (CXCL1) and chemokine (C-C motif) ligand 5 (CCL5) [85, 86]. Moreover, PPAR-*γ* inhibited the maturation of DC and attenuated the expression of CD1a, CD40, CD80, CD83, and chemokine (C-C motif) receptor 7 (CCR7) [85, 88, 90]. These results indicate that PPAR-*γ* activation by synthetic ligands reduced the ability of DC to stimulate lymphocyte proliferation and to prime antigen-specific T cell responses.

## 6. PPAR-**γ** and Neutrophils

Neutrophils, as well as macrophages, lymphocytes, and DC, also play crucial roles in atherogenesis [91, 92]. Neutrophils and their mediators have been detected in mouse and human atherosclerotic lesions [93–95]. An increased number of circulating neutrophils are also observed in pathological conditions such as hyperlipidemia. Neutrophils are recruited into atherosclerotic lesions via specific chemokine receptors, including CCR1, 2, 5, and CXCR2 [96]. OxLDL may induce the transmigration of neutrophils and release of ROS and granule proteins from neutrophils, which trigger monocyte recruitment and extravasation directly or indirectly through upregulation of adhesion molecules on endothelial cells. In addition, apoptotic neutrophils sustain monocyte recruitment via various find-me and eat-me signals [97]. Thus, neutrophils could provide a chronic inflammation trigger sustaining atherogenesis.

Several lines of evidence have demonstrated the presence of PPAR-*γ* in neutrophils, and have shown a suppressive effect of PPAR-*γ* activation by endogenous and synthetic ligands on neutrophil infiltration in various animal models of inflammation [98–103]. A recent study by Napimoga et al. [104] reported that administration of 15d-PGJ_2_, an endogenous PPAR-*γ* ligand, decreased leukocyte rolling and adhesion to inflamed mesenteric tissue by a mechanism dependent on NO. Specifically, pharmacological inhibitors of NO synthase (NOS) abrogated the 15d-PGJ_2_-mediated suppression of neutrophil migration to the inflammatory site. Moreover, inducible NOS^−/−^ mice were not susceptible to 15d-PGJ_2_-mediated suppression of neutrophil migration to inflammatory sites compared with their wild type. In addition, 15d-PGJ_2_-mediated suppression of neutrophil migration appeared to be independent of the production of cytokines and chemokines, since their production was not significantly affected in the carrageenan-injected peritoneal cavity. These findings demonstrated that 15d-PGJ_2_ suppresses inflammation-initiated neutrophil migration in a mechanism dependent on NO production in mesenteric tissue. However, the detailed role of neutrophil regulation by PPAR-*γ* ligands in atherosclerosis remains to be elucidated and further studies are needed.

## 7. Recent Concern for Cardiovascular Risks of PPAR-**γ** Agonists

As described above, PPAR-*γ* activation is expected as a therapeutic target for improving cardiovascular risk factors. However, its safety is controversial in clinical use, since several reports pointed out an increase in risk of ischemic cardiovascular events with PPAR-*γ* agonists. Meta-analysis of randomized controlled trials has suggested that rosiglitazone, one of TZD, increased risk of ischemic cardiovascular events [105, 106]. In contrast, meta-analysis of clinical trials of another TZD, pioglitazone has also reported the possibility of an ischemic cardiovascular benefit by pioglitazone [107]; however, both TZDs are reported to increase the risk of congestive heart failure [108]. Recently, meta-analysis of observational studies that directly compared the risk of cardiovascular outcomes for rosiglitazone and pioglitazone among patients with T2DM has demonstrated that the use of rosiglitazone was associated with significantly higher odds of congestive heart failure, myocardial infarction, and death compared with that of pioglitazone [109]. However, whether any meaningful difference exists in the magnitude of risk between two TZDs is still unclear. The European Medicines Agency has recommended the suspension of marketing authorization for rosiglitazone, whereas the US Food and Drug Administration has allowed the continued marketing of rosiglitazone with additional restrictions [110]. Further studies are required to understand these contradictory effects of PPAR-*γ* agonists in the future.

## 8. Conclusion

Vascular inflammation-induced atherosclerosis is one of the most worrying common problems throughout the world. As described above, PPAR-*γ* has a wide range of roles in the pathogenesis and progression of atherosclerosis via regulation of inflammatory cells, including monocytes/macrophages, lymphocytes, dendritic cells, and neutrophils. Although regulation of PPAR-*γ* activity may not alter the underlying cause of the disease, it may regulate pathological conditions, resulting in clinical benefit. Several recent experimental and clinical findings have supported the potential utility of regulating PPAR-*γ* activity as a therapeutic approach for atherosclerosis. The roles of PPAR-*γ* regulation still represent huge unmet challenges in therapeutic interventions. Further accumulation of experimental and clinical evidence on the relationship between PPAR-*γ* and vascular inflammation may contribute to solving this problem.

## Figures and Tables

**Table 1:** Endogenous and synthetic ligands for PPAR-*γ* and genes for PPAR-*γ* related coactivator.

Ligands for PPAR-*γ*		Genes for PPAR-*γ* related co-activator
Endogenous ligands	Synthetic ligands
Unsaturated fatty acids	Rosiglitazone	CBP/p300
15-deoxy-Δ^12, 14^-prostaglandin J_2_	Pioglitazone	SRC-1
15-HETE	Troglitazone	SRC-2
9-HODE	Ciglitazone	SRC-3
13-HODE	Tyrosine derivatives	PGC-1*α*
Oxidized LDL	Farglitazar	PGC-1*β*
	GW7845	PBP
		PRIP
		PRIC285
		BAF60c

BAF60c: BRG1/Brm-associated factor of 60 kDA subunit of c; CBP: cyclic-AMP responsive element binding protein (CREB)-binding protein; HETE: hydroxyeicosatetraenoic acid; HODE: hydroxyoctadecadienoic acid; LDL: low-density lipoprotein; PBP: PPAR-binding protein; PGC: PPAR-*γ* coactivator; PPAR: peroxisome proliferator-activated receptor; PRIC: PPAR-*α*-interacting cofactor; PRIP: PPAR interacting protein; SRC: steroid receptor coactivator.
